# ADAM9 Expression Is Associate with Glioma Tumor Grade and Histological Type, and Acts as a Prognostic Factor in Lower-Grade Gliomas

**DOI:** 10.3390/ijms17091276

**Published:** 2016-08-26

**Authors:** Xing Fan, Yongheng Wang, Chuanbao Zhang, Li Liu, Sen Yang, Yinyan Wang, Xing Liu, Zenghui Qian, Shengyu Fang, Hui Qiao, Tao Jiang

**Affiliations:** 1 Beijing Neurosurgical Institute, Capital Medical University, Beijing 100050, China; xingkongyaoxiang@163.com (X.F.); 13031887878@163.com (Y.W.); chuanbao123@139.com (C.Z.); tiantanyinyan@126.com (Y.W.); 15846591696@126.com (X.L.); qianzenghui1987@163.com (Z.Q.); fangtuo1@aliyun.com (S.F.); 2 Department of Neurosurgery, Qinhuangdao First Hospital, Qinhuangdao 066000, China; 3 Department of Ophthalmology, Qinhuangdao First Hospital, Qinhuangdao 066000, China; 15603377991@163.com; 4 Department of Radiotherapy, Qinhuangdao First Hospital, Qinhuangdao 066000, China; yangsen929@163.com; 5 Department of Neurosurgery, Beijing Tiantan Hospital, Capital Medical University, Beijing100050, China

**Keywords:** A disintegrin and metalloproteinases 9 (ADAM9), glioma, histological type, tumor grade, prognosis

## Abstract

The A disintegrin and metalloproteinase 9 (ADAM9) protein has been suggested to promote carcinoma invasion and appears to be overexpressed in various human cancers. However, its role has rarely been investigated in gliomas and, thus, in the current study we have evaluated ADAM9 expression in gliomas and examined the relevance of its expression in the prognosis of glioma patients. Clinical characteristics, RNA sequence data, and the case follow-ups were reviewed for 303 patients who had histological, confirmed gliomas. The ADAM9 expression between lower-grade glioma (LGG) and glioblastoma (GBM) patients was compared and its association with progression-free survival (PFS) and overall survival (OS) was assessed to evaluate its prognostic value. Our data suggested that GBM patients had significantly higher expression of ADAM9 in comparison to LGG patients (*p* < 0.001, *t*-test). In addition, among the LGG patients, aggressive astrocytic tumors displayed significantly higher ADAM9 expression than oligodendroglial tumors (*p* < 0.001, *t*-test). Moreover, high ADAM9 expression also correlated with poor clinical outcome (*p* < 0.001 and *p* < 0.001, log-rank test, for PFS and OS, respectively) in LGG patients. Further, multivariate analysis suggested ADAM9 expression to be an independent marker of poor survival (*p* = 0.002 and *p* = 0.003, for PFS and OS, respectively). These results suggest that ADAM9 mRNA expression is associated with tumor grade and histological type in gliomas and can serve as an independent prognostic factor, specifically in LGG patients.

## 1. Introduction

Gliomas are the most common and aggressive primary brain tumors in adults and account for over 70% of the total cases [1,2]. Based on 2007 World Health Organization (WHO) classification, gliomas are classified into four grades, from WHO grade I to WHO grade IV: WHO grade I tumors are often stable and can be cured by surgery; however, diffuse low-grade and intermediate-grade gliomas (WHO grades II and III) are generally infiltrative and together constitute lower-grade gliomas (LGG) [3]. The glioblastoma (WHO grade IV) is the most lethal and aggressive form among all grades. Additionally, gliomas can be divided into two subtypes based on their histological appearance: astrocytic tumors and oligodendroglial tumors, including both pure oligodendroglial tumors and mixed oligoastrocytic tumors [4]. Oligodendroglial tumors are often less aggressive and patients with such tumors tends to have better health and survival. However, astrocytic tumors, especially the most frequent and lethal type, glioblastoma (GBM), have very poor survival [5]. So far, gliomas represent a great challenge for clinicians due to their dismal prognosis. Thus, in recent decades, clinicians and scientists have started to pay more attention to identifying specific tumor-related molecular markers, which then in concert with pathological classification can be used for the designing of individualized treatments [6,7]. These types of scientific efforts have recently led to substantial progress and different tumor-specific molecular changes, such as isocitrate dehydrogenase 1 (IDH1) mutation [8], 1p/19q co-deletion [9], O6-methylguanine DNA methyltransferase promoter methylation [10], telomerase reverse transcriptase promoter mutation [11], epithelial growth factor receptor amplification [12] and a few others, have been identified as predictive and prognostic indicators and/or therapeutic targets for glioma patients. However, considering the complexity of the human genome, additional molecular alterations associated with the pathogenesis and development of glioma must be there and still need to be elucidated. Moreover, the identification of new biomarkers can provide more options to confidently predict the survival and/or response of individualized treatment therapy in gliomas patients.

The A disintegrin and metalloproteinase (ADAMs) family members are multifunctional and membrane-bound cell surface glycoproteins belonging to the zinc protease superfamily and play important roles in regulating the cell phenotype through their effects on many cellular processes including cell growth, differentiation, and motility [13]. The ADAM family proteins are usually considered to play a fundamental role in controlling homeostasis and development, and their aberrant expression is thought to be related with pathological states, including cancer [14,15,16], inflammation [17], diabetes [18] and Alzheimer’s disease [19]. ADAM9, a member of this family, has been suggested to promote carcinoma invasion [20], and seems to be markedly up-regulated in many human cancers [21,22,23,24,25,26,27,28]. In addition, it has also been indicated that ADAM9 might potentially contribute to the pathogenesis of human cancers, and can potentially be a good therapeutic target for developing anti-cancer drugs [29].

Surprisingly, ADAM9 has rarely been investigated in gliomas, and to our knowledge, very little information is available so far on its role in gliomas. Therefore, in the current study, we have evaluated the ADAM9 mRNA expression in gliomas, using information from 303 glioma patients based on their RNA sequence data. Additionally, we have also tried to evaluate its prognostic significance in different grades of gliomas. 

## 2. Results

### 2.1. Patient Characteristics

The clinical and RNA-seq data were obtained from all 303 patients. Among these, 170 patients were characterized to have LGGs (including 105 WHO grade II and 65 WHO grade III gliomas), and 133 were diagnosed to have GBM (WHO grade IV). At the time of diagnosis, the median age of the subjects was 43 years (range eight to 81 years). The baseline characteristics of all patients are summarized in Table 1.

### 2.2. Analysis of ADAM9 mRNA Expression in Glioma Patients

**Figure 1:**
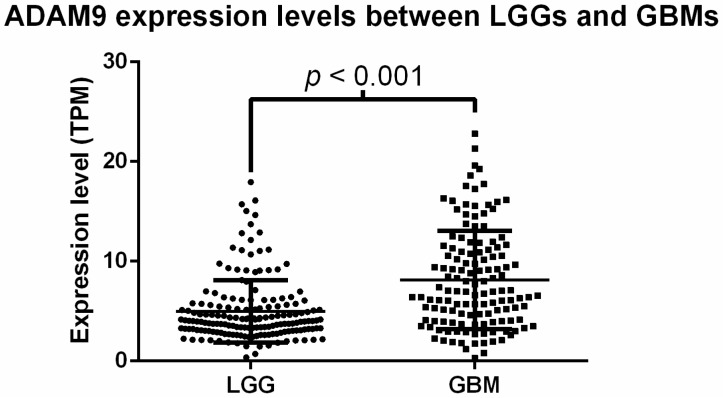
Comparison of ADAM9 mRNA expression levels between LGG and GBM tumor samples. The LGG tumor samples displayed an average of 4.098 ± 2.132 TPM units of ADAM9 mRNA expression, while GBM tumor samples displayed an average of 8.139 ± 4.922 TPM units of ADAM9 mRNA expression. The difference was significant between the two subtypes, *p* < 0.001, *t*-test. TPM, transcripts per million; LGG, lower-grade glioma; GBM, glioblastoma.

**Figure 2:**
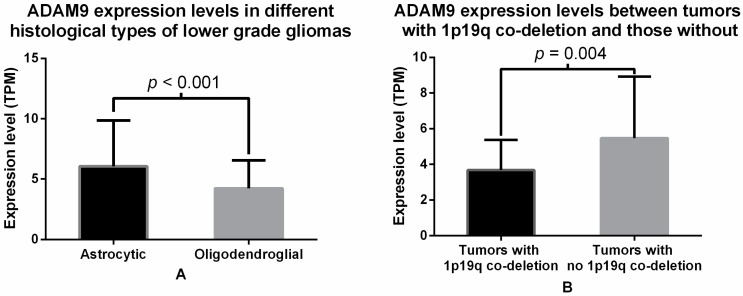
(**A**) Comparison of ADAM9 mRNA expression levels between LGG tumor samples with different histological type. Black bar represents the average ADAM9 mRNA expression from astrocytic tumors (6.051 ± 0.460 TPM units), while white bar represents ADAM9 mRNA expression in oligodendroglial tumors (4.228 ± 0.231, TPM units). The difference is significant with a *p*-value of <0.001, *t*-test; (**B**) Comparison of ADAM9 mRNA expression levels between LGG tumor samples with and without 1p/19q co-deletion. Black bar represents the average ADAM9 mRNA expression in tumors with 1p/19q co-deletion (3.386 ± 0.289 TPM units), while white bar represents ADAM9 mRNA expression in tumors with no 1p/19q co-deletion (5.476 ± 0.332, TPM units). The difference is significant with *p*-value of 0.004, *t*-test.

The gene expressions of 22 members of the ADAM family were compared between patients with different grades of gliomas. Among these, 14 genes showed a statistically significant difference in the mRNA expression between different grades of gliomas, including ADAM9 (Supplementary Table S1). ADAM9 mRNA expression was higher in GBM patient samples (8.139 ± 4.922 transcripts per million, TPM, units) as compared to LGG patients (4.098 ± 2.132 TPM units), and this difference was significant (*p* < 0.001, *t*-test, Figure 1). Next, the potential correlation between ADAM9 expression and clinical characteristics was examined using Chi-square analysis (Table 2). We observed a significant association between ADAM9 expression and histological type in patients with lower-grade gliomas (*p* < 0.001, Chi-square test). Further analysis of the association between ADAM9 expression and different histological types of LGG revealed that astrocytic tumors had significantly higher expression than oligodendroglial tumors (6.051 ± 0.460 vs. 4.228 ± 0.231, *p* < 0.001, *t*-test, Figure 2A). Additionally, we also found that the ADAM9 expression level was significantly associated with 1p/19q co-deletion in patients with lower-grade gliomas (*p* = 0.002, Chi-square test). Student’s *t*-test, used to determine this association, more specifically showed that ADAM9 expression was significantly lower in LGG patients with the 1p/19q co-deletion.

### 2.3. Correlation between ADAM9 mRNA Expression and Patient Survival

**Figure 3:**
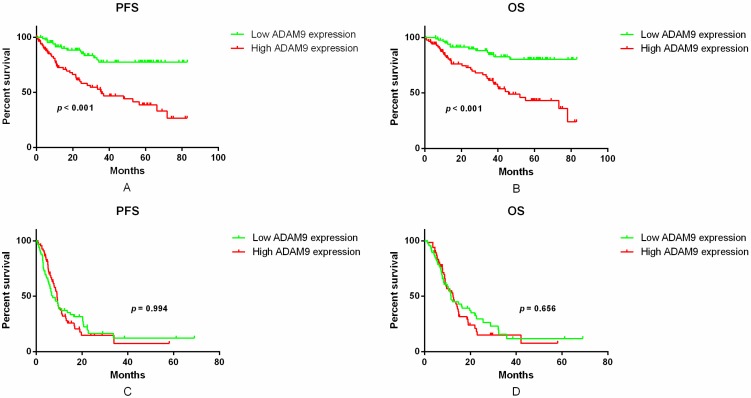
Kaplan–Meier survival analysis of different grades of glioma patients based on ADAM9 expression. (**A**) Comparison of the PFS between ADAM9 high and low expression group in patients with LGG tumors (*p* < 0.001, log-rank test); (**B**) Comparison of the OS between ADAM9 high and low expression group in patients with LGG tumors (*p* < 0.001, log-rank test); (**C**) Comparison of the PFS between ADAM9 high and low expression group in patients with GBMs (*p* = 0.994, log-rank test); (**D**) Comparison of the OS between ADAM9 high and low expression group in patients with GBMs (*p* = 0.656, log-rank test). PFS, Progression-free survival; OS, overall survival; LGG, lower-grade glioma; GBM, glioblastoma.

To determine the association between ADAM9 mRNA expression and clinical outcomes, the data from the cohort of 303 glioma patients was analyzed. The mean follow-up time of the glioma patients was 25.8 ± 21.8 months (range 0.7–83.0 months; median, 17.2 months). In total, 141 patients died during the follow-up time (65.2% males). The Kaplan–Meier analysis showed that there was a significant difference in both progression-free survival (PFS, *p* < 0.001, log-rank test, Figure 3A) and overall survival (OS, *p* < 0.001, log-rank test, Figure 3B) between the LGG patients having high or low ADAM9 expression. The LGG patients with low ADAM9 expression were observed to have a better survival than those with high ADAM9 expression. In contrast, no significant differences were observed with respect to either PFS or OS in GBM patients, based on ADAM9 expression levels (*p* = 0.994 and 0.656, log-rank test, PFS and OS, respectively, Figure 3C,D). This result indicated that ADAM9 expression can serve as a potential prognostic factor for at least LGG patients.

In addition, a multivariate progression analysis was performed to test the independent value of each variable predicting PFS and OS in patients with lower-grade gliomas, using the Cox proportional hazard model. It was observed that high ADAM9 expression appeared to be a predictor for poor clinical outcomes in the LGG patient population (*p* < 0.001 and *p* = 0.001 for PFS and OS, respectively, Table 3). Moreover, we also identified through this analysis that histological type could be a predictive factor for LGG prognosis (*p* = 0.002 and 0.004 for PFS and OS, respectively, Table 3) and age could be a predictive factor for PFS in LGG patients (*p* = 0.031, Table 3). 

## 3. Discussion

Our study analyzed for the first time the expression of ADAM9 in a large number of human glioma patients. The retrospective analysis of clinical and RNA-seq data pertaining to 303 histologically confirmed glioma patients was performed. ADAM9 mRNA expression was observed to be associated with tumor grade and histological type in glioma patients. Moreover, it seems that low ADAM9 mRNA expression may serve as an independent prognostic factor for better clinical outcomes in LGG patients. 

Cancer invasion and metastasis have complex genetic and biochemical determinants and are still not completely understood in terms of their molecular mechanism. Both of these are multistep events that include angiogenesis, local invasion, cell migration, extravasation and tumor growth, and may share similar mechanisms. ADAM9, also called metalloprotease disintegrin cysteine-rich protein-9 or meltrin γ, has originally been described as a membrane-anchored cell surface protein that is widely expressed in human tissues [30]. Its up-regulation has been reported in various human cancers including breast [21,23], pancreatic [22], gastric [24], renal [25], and prostate [23]. It has been previously reported that a secreted form of ADAM9 potently promotes cancer cell invasion by modulating tumor-stromal interactions [20]. Also, another recent study has shown that ADAM9 transcripts are alternatively spliced to express secreted and transmembrane isoforms. The secreted isoform promotes breast cancer cell migration in a manner dependent upon its metalloproteinase activity, while the transmembrane isoform suppresses cell migration independent of such activity [31]. Additionally, an elevated ADAM9 expression level was also found in liver metastases from colon carcinomas and brain metastases from non-small cell lung cancer [32,33]. All these previous reports above suggested that ADAM9 may potentially play an important role in cancer invasion and metastasis. 

The higher order of invasiveness in glioma is an important reason for its poor prognosis, and molecular changes involved in this invasiveness can turn out to be potentially therapeutic targets. Secretion of proteases in general has been shown to be associated with the remodeling of the extracellular environment that in turn can enhance the motility of tumor cells. Many families of proteases, such as matrix metalloproteinases, are implicated in the invasive process of brain tumors [34,35]. Consistent with these published reports, we in this study identified that GBMs, the most aggressive subtype, had significantly higher expression of ADAM9 compared to LGGs. Also, a significant difference in the ADAM9 mRNA expression was observed between LGG patients with different histological characteristics, and astrocytic tumors, which are more aggressive, had significantly higher ADAM9 mRNA expression than oligodendroglial tumors. It has been widely accepted that 1p/19q co-deletion is the most established marker in predicting better prognosis in LGG and it also correlate closely with the oligodendroglial component of tumors [36]. In the current study, we also found that tumors with 1p/19q co-deletion have significantly lower ADAM9 expression. This result was consistent with the association we found between ADAM9 expression and histological type. Thus, this data did indicate that ADAM9 might play an important role in the invasion of gliomas, just like its role in other human cancers, and have the potential to serve as a predictor of prognosis in LGG. 

In addition to ADAM9, several other members of the ADAM family have also been implicated in tumor growth and invasiveness of gliomas, such as ADAM10, ADAM12 and ADAM17 [37,38,39]. However, ADAM9 was rarely investigated in gliomas, and thus far its precise function was still unclear. There was a previous study which suggested that ADAM9 expression may contribute to glioblastoma invasion in U87 cells [40]. Another recent study identified that ADAM9, through potentially regulating the activity of tenascin-C protein, might stimulate the invasiveness of brain tumor-initiating cells [41]. Except for these studies, there are not very many studies directly linking ADAM9 with glioma progression. However, ADAM9, through other mechanisms, has been linked with the regulation of the tumor growth of various other human cancers; for example, the silencing of ADAM9 has been shown to reduce tumor cell proliferation and the migration of esophageal squamous cell carcinoma cells by inhibiting epidermal growth factor receptor (EGFR)/protein kinase B (AKT) signaling [42]. ADAM9 can also function as an adhesion molecule by interacting with an αvβ5 integrin on myeloma cells [43]. Thus, it would be important to understand whether these additional mechanisms do play any role in the contribution of ADAM9 to the progression of glioma and would require further investigation. 

The association of high ADAM9 expression with poor clinical outcomes has been broadly investigated and reported in various other human malignancies. For instance, ADAM9 protein expression has been shown to significantly associate with poor patient survival in ductal adenocarcinoma and renal cell cancer patients [22,25]. Additionally, ADAM9 protein expression was also reported to be significantly associated with shortened prostate-specific antigen relapse-free survival in prostate cancer [26]. Importantly, a recent study showed that high ADAM9 expression was an independent factor linked with shortened survival and has been proposed to serve as a predictive biomarker for the selection of non-small cell lung cancer patients eligible for postoperative adjuvant chemotherapy treatment [28]. Concordant with these observations in other human cancers, we also observed that high ADAM9 mRNA expression was associated with poor clinical outcomes and thus we speculate that it could serve as an independent prognostic factor in LGGs. To our knowledge, this is the first study which demonstrated that ADAM9 overexpression can act as a prognostic factor for poor clinical outcomes in gliomas. Notably, a recent study has reported that fisetin, a natural flavonoid widely distributed in plants, could suppress ADAM9 protein and mRNA expression and inhibit the migration and invasion of glioma cancer cells [44]. This finding reinforces the idea that ADAM9 could also be a potential target for improved individualized treatment. 

## 4. Materials and Methods

### 4.1. Patients and Tissue Samples

A total of 303 patients were enrolled in our study from January 2007 to February 2013. All the patients had histological confirmed gliomas and information about their RNA sequence data. The histological diagnosis were performed independently by two experienced neuropathologists according to the 2007 WHO classification [4]. All patients had no history of detectable precursor lesions prior to admission and underwent surgical resection in the Department of Neurosurgery, Beijing Tiantan Hospital. Also no patient died of unexpected events or other diseases during evaluation. Clinical information about all these patients was obtained from the institutional database. This study was approved by the ethics committee of Beijing Tiantan Hospital and the written informed consent was obtained from all the patients. Tissue samples were immediately snap-frozen in liquid nitrogen after resection. The percentage of tumor cells was assessed by examining the cell morphology by hematoxylin and eosin (HE) staining of the frozen sections. Only samples with ≥80% of the tumor cell population were selected for the study in order to reduce the influence of contamination.

### 4.2. RNA-Seq Library Preparation and Quality Control

RNA-seq library was constructed as described in our previous studies [45,46]. Briefly, RNA extraction was carried out using RNeasy Mini Kit (Qiagen, Duesseldorf, Germany) according to the manufacturer’s instructions. The frozen tissue sample was disrupted and homogenized using QIAshredder column (Qiagen, Duesseldorf, Germany). Subsequently, a 2100 Bioanalyzer (Agilent, Santa Clara, CA, USA) was used to assess the RNA quality and only samples with RNA Integrity of over 7.0, were used to construct the sequencing library. After PCR enrichment and purification of the adapter-ligated fragments, quantitative PCR was performed by using Applied Biosystems 7500 instrument (Thermo Fisher Scientific, MA, USA) with Illumina’s small RNA primer set QP1 5′-AATGATACGGCGACCACCGA-3′ and QP2 5′-CAAGCAGAAGACGGCATACGAGA-3′. The length of the resulting cDNA fragments was determined by the Agilent 2100 Bioanalyzer. The libraries were subsequently sequenced on the Illumina HiSeq 2000 platform (Ilumina, San Diego, CA, USA) using 101 bp pair-end sequencing strategy. Image data was converted into sequence data using base calling software (Illumina pipeline CASAVA v1.8.2, CA, USA) and then subjected to standard quality control criteria. Sequence reads which fit any of the following parameters were excluded: (1) the reads aligning to adaptors or primers with no more than two mismatches; (2) the reads with over 10% unknown bases; and (3) the reads with over 50% of the bases with a quality value of less than five in one read. After quality control, 1308.3 giga base pairs (94.4%) of the filtered reads were analyzed.

### 4.3. Read Mapping and Expression Analysis of RefSeq Genes

RNA expression levels were determined by mapping the reads to the RefSeq-RNA reference sequence set Hg 19 (RNA sequences, GRCh37), which was downloaded from the UCSC Genome Browser (available online: http://genome.ucsc.edu). The expression levels of genes were evaluated in units of TPM, which is a superior measure to reads per kilobase transcriptome per million when comparing different samples [47]. The influence of varying gene lengths and sequencing discrepancies were removed using the TPM method while calculating the gene expression. Accordingly, the estimated gene expression can be directly compared between different samples. 

### 4.4. Detection of IDH1 Mutation and 1p/19q Co-Deletion

Genomic DNA was extracted from frozen tissues by the QIAamp DNA Mini Kit (Qiagen, Dusseldorf, Germany) according to the manufacturer’s protocol. Then DNA concentration and quality were measured subsequently using the Nano-Drop ND-1000 spectrophotometer (NanoDrop Technologies, Houston, TX, USA). Pyrosequencing of IDH1 mutation was supported by Gene-tech (Shanghai, China) and performed on the Pyro-Mark Q96 ID System (Qiagen, Dusseldorf, Germany). The primers (forward) 5′-GCTTGTGAGTGGATGGGTAAAAC-3′ and (reverse) 5′-Biotin-TTGCCAACATGACTTACTTGATC-3′ were used in PCR amplification. The primer 5’-TGGATGGGTAAAACCT-3′ was used in pyrosequencing. Moreover, 1p/19q loss was detected by fluorescence in situ hybridization using LSI probe sets 1p36/1q25 and 19q13/19p13 (spectrum orange-labeled 1p36 and 19q13 probes; spectrum green-labeled 1q25 and 19p13 probes; Vysis), and then evaluated in over 200 non-overlapping nuclei.

### 4.5. Statistical Analysis

Statistical analyses were performed using SPSS software version 16.0 (SPSS Inc., Chicago, IL, USA). The *p*-value of <0.05 was considered statistically significant. The mean expression levels were compared using student’s *t*-test between patients with different grades of gliomas. For further analysis, patients with LGGs and GBMs were respectively subdivided into two subgroups based on ADAM9 expression levels (cut off at 50% of the entire group). Univariate analysis was performed using chi-square test for dichotomous clinical variables. Kaplan-Meier analysis (log-rank test) was used to evaluate predictive value of ADAM9 expression for progression-free survival (PFS) and overall survival (OS) in patients with different grades of gliomas. Censored events for OS were defined as deaths of patients without evidence of any other disease, while censored events for PFS were defined as tumor recurrences as diagnosed by MR images. At last, Cox’s proportional hazards model was used to determine the independent association of prognostic variables with PFS and OS.

## 5. Conclusions

Our study demonstrated that ADAM9 mRNA expression was associated with tumor grade and histological type in gliomas. Furthermore, a significant association between high ADAM9 expression and poor clinical outcome in LGG patients was observed. Our results indicated that ADAM9 expression could serve as a prognostic marker in LGG patients along with being a potential therapeutic target. Further studies would be required to elucidate the precise function of ADAM9 protein in gliomas, in addition to understanding the contribution/role of other ADAM family members. 

## Figures and Tables

**Table 1:** Clinical characteristics of 303 glioma patients.

Variables	Lower-Grade Glioma	Glioblastoma
Numbers	170	133
Median age (range)	39 (10–75)	49 (8–81)
Sex (male)	103	86
Pathology (Astrocytic)	69	-
IDH1 mutational status	-	-
IDH1 mutation	100	18
IDH1 wild-type	34	78
1p/19q status	-	-
1p/19q co-deletion	34	2
No 1p/19q co-deletion	108	106

**Table 2:** Association between ADAM9 expression level and clinical characteristics *.

Variables	Lower-Grade Glioma			Glioblastoma		
	Low ADAM9 Expression	High ADAM9 Expression	*p*-Value	Low ADAM9 Expression	High ADAM9 Expression	*p*-Value
Age > 40	36	39	0.643	43	56	0.015
Sex (male)	46	57	0.084	43	43	0.907
Pathology (Astrocytic)	24	45	<0.001	-	-	-
IDH1 mutation	54	46	0.686	11	7	0.253
IDH1 wild-type	17	17	-	36	42	-
1p/19q co-deletion	25	9	0.002	0	2	0.496
No 1p/19q co-deletion	47	61	-	53	53	-

* Results of Chi-square test.

**Table 3:** Multivariate predictors of PFS and OS for patients with LGGs *.

Variables	PFS			OS		
	Risk Ratio	95% CI	*p*-Value	Risk Ratio	95% CI	*p*-Value
High ADAM9 expression	2.682	1.432–5.022	0.002	2.789	1.419–5.482	0.003
Age > 40	1.863	1.065–3.169	0.022	1.740	0.986–3.072	0.056
Sex (male)	0.779	0.452–1.343	0.291	0.870	0.486–1.558	0.639
Pathology (Astrocytic)	2.334	1.315–4.142	0.004	2.221	1.211–4.075	0.010

* Results of Cox regression analysis.

## Supplementary Materials

The following are available online at www.mdpi.com/1422-0067/17/9/1276/s1.

## Abbreviations

ADAM9	A disintegrin and metalloproteinases 9
LGG	lower-grade glioma
GBM	glioblastoma
IDH1	isocitrate dehydrogenase 1
PFS	progression-free survival
OS	overall survival
HE	hematoxylin and eosin
TPM	transcripts per million
