# Supplementary material for: ADAM9 Expression Is Associate with Glioma Tumor Grade and Histological Type, and Acts as a Prognostic Factor in Lower-Grade Gliomas
Source: Int J Mol Sci. 2016 Aug 26;17(9):1276. doi: 10.3390/ijms17091276 (PMC5037653; doi:10.3390/ijms17091276)
Supplement: Supplementary file 1 [file ijms-17-01276-s001.pdf]

Supplementary Materials: ADAM9 Expression Is Associate with Glioma Tumor Grade and Histological Type, and Acts as a Prognostic Factor in Lower-Grade Gliomas

Xing Fan, Yongheng Wang, Chuanbao Zhang, Li Liu, Sen Yang, Yinyan Wang, Xing Liu, Zenghui Qian, Shengyu Fang, Hui Qiao and Tao Jiang

Table S1. Comparison of the gene expression of 22 members in ADAM family between LGGs and GBMs patients.

Gene Symbol	*p*-Value	Pathological Functions in Human Cancers
ADAM1A	0.056	Uncharacterized
ADAM3A	0.408	Uncharacterized
ADAM5	0.223	Uncharacterized
ADAM6	<0.001	Uncharacterized
ADAM7	0.179	Uncharacterized
ADAM8	0.002	The protein encoded by this gene may be involved in cell adhesion during neurodegeneration (provided by RefSeq, August 2009)<br>Up-regulated in gliomas and associated with local invasion [14]
ADAM9	<0.001	As we described in the paper
ADAM10	0.552	This gene encodes an ADAM family member that cleaves many proteins including TNF-alpha and E-cadherin (provided by RefSeq, February 2016)
ADAM11	<0.001	Represents a candidate tumor suppressor gene for human breast cancer (provided by RefSeq, January 2016)
ADAM12	<0.001	Up-regulated by TGF-β in human activated hepatic stellate cells and associate with tumor aggressiveness and progression in liver cancer [32]
ADAM15	<0.001	This protein may function in cell-cell adhesion as well as in cellular signaling (provided by RefSeq, July 2008)
ADAM17	0.234	Functions in the processing of numerous other substrates, including cell adhesion proteins, cytokine and growth factor receptors and epidermal growth factor receptor ligands (provided by RefSeq, February 2016)
ADAM18	0.082	Uncharacterized
ADAM19	<0.001	An active metalloproteinase which may be involved in cell migration, cell adhesion, cell-cell and cell-matrix interactions, and signal transduction (provided by RefSeq, May 2013)<br>Up-regulated in gliomas and associated with local invasion [14]
ADAM20	0.005	Uncharacterized
ADAM21	0.019	Uncharacterized
ADAM22	<0.001	Inhibits cellular proliferation of glioma-derived astrocytes [48]
ADAM23	0.014	It is reported that inactivation of this gene is associated with tumorigenesis in human cancers (provided by RefSeq, May 2013)
ADAM28	0.429	Involved in cancer cell proliferation, invasion and metastasis [49]
ADAM30	0.073	Uncharacterized
ADAM32	<0.001	Uncharacterized
ADAM33	<0.001	A novel regulatory mechanism in the IL-18-secreted process in gastric cancer [50]
